# Antibiotic Use Among Children Requiring Respiratory Support in Intensive Care Unit (ICU) from Sofia, Bulgaria: A Single-Center Retrospective Experience

**DOI:** 10.3390/antibiotics15020225

**Published:** 2026-02-19

**Authors:** Lilia Bozadzhieva, Dimitrinka Miteva, Lyubomila Ilarionova, Tania Teneva, Blagomir Zdravkov, Guergana Petrova

**Affiliations:** 1 Pediatric Intensive Care Unit, University Hospital for Active Treatment of Children’s Diseases “Professor Ivan Mitev”, Akademik Ivan Evstatiev Geshov Blvd 11, 1431 Sofia, Bulgaria; lilita.chem@gmail.com (L.B.); lyubomila.dimitrova@gmail.com (L.I.); tania_teneva@yahoo.com (T.T.); blagomir.zdravkov@gmail.com (B.Z.); 2 Department of Pediatric, Medical University of Sofia, Akademik Ivan Evstatiev Geshov Blvd 11, 1431 Sofia, Bulgaria; dimi_sm@abv.bg; 3 Pediatric Clinic, University Hospital “Alexandrovska”, 1 Georgi Sofiyski Blvd, 1431 Sofia, Bulgaria

**Keywords:** non-invasive ventilation, invasive mechanical ventilation, antibiotic use, ventilator-associated pneumonia, respiratory failure

## Abstract

Antibiotic use in critically ill children requiring respiratory support remains controversial, particularly in the absence of standardized guidelines for patients managed with non-invasive ventilation (NIV). Evidence in this area remains limited, and real-world data are therefore valuable. **Objective:** This retrospective single-center study aimed to describe antibiotic prescribing patterns and infectious outcomes in pediatric patients admitted to the intensive care unit (PICU) with respiratory failure, according to the type of respiratory support. **Methods:** Children aged 0–17 years admitted between January 2021 and February 2025 who required oxygen supplementation, NIV, or invasive mechanical ventilation (IMV) were included. Demographic characteristics, underlying conditions, infectious complications, antibiotic exposure, length of PICU stay, and outcomes were analyzed using descriptive statistics and univariate comparisons. **Results:** Eighty-nine patients were included. Ventilator-associated pneumonia (VAP) occurred exclusively in patients receiving IMV, and infection complications were observed more in this group compared to those receiving NIV (*p* = 0.005). *Pseudomonas aeruginosa* was the most frequently isolated pathogen. Antibiotics were administered in 82% of patients, with no significant association between the respiratory support and initiation of antibiotic therapy (*p* = 0.195). A higher number of antibiotics was administered in patients receiving IMV compared with those receiving oxygen therapy alone. **Conclusions**: Antibiotic use in children requiring respiratory support in the PICU was common and appears to be driven primarily by underlying disease and illness severity rather than by the ventilation modality alone. Infections specific to invasive ventilation, such as VAP, were more frequent in patients receiving IMV, while infection-related outcomes in non-invasive groups should be interpreted cautiously due to differences in diagnostic definitions. These findings are descriptive and hypothesis-generating and highlight the need for prospective multicenter studies to create evidence-based antibiotic stewardship strategies in pediatric critical care.

## 1. Introduction

In emergency settings, mechanical respiratory support has been shown to increase survival rates and reduce mortality [1]. However, invasive mechanical ventilation (IMV) carries significant risks and potential complications [2]. Respiratory infections, specifically ventilator-associated pneumonia (VAP) and ventilator-associated tracheobronchitis (VAT), are well-documented drivers of poor outcomes in intubated patients [3,4] and remain a critical clinical challenge.

The use of non-invasive mechanical ventilation (NIV) in pediatric critical care has expanded significantly over the last two decades, establishing it as a primary intervention for respiratory support. By assisting ventilation without an invasive airway, NIV aims to circumvent the morbidity and mortality associated with IMV [5]. It represents an effective and safe alternative for specific pediatric populations, including those with acute respiratory failure and chronic conditions requiring long-term assistance. Advantages of NIV over IMV include reduced airway trauma, lower sedation requirements, shorter durations of ventilator support, fewer nosocomial infections, and decreased pediatric intensive care unit (PICU) length of stay [6,7,8].

Despite the increasing adoption of NIV in pediatric critical care, no standardized or evidence-based guidelines exist for antibiotic use in children receiving NIV in the PICU, leading to considerable variation in clinical practice. Published pediatric data on the role of antibiotics in preventing or treating infection-related complications in this population are scarce, and the benefit of antibiotic therapy for the prevention of lower respiratory tract infections remains controversial. A recent prospective study of over 11,000 bronchiolitis patients requiring ICU respiratory support found that early antibiotic use did not improve clinical outcomes such as ICU length of stay or the need for intubation [9]. While these findings are unsurprising given the viral etiology of bronchiolitis, up to 54% of children in that study did receive antibiotics on the first ICU day [9]. This highlights the challenge of distinguishing bacterial from viral infections in pediatrics and underscores that antibiotics should be reserved for confirmed or highly suspected bacterial infections. Existing data for VAT, VAP, and antibiotic use primarily derive from adult populations. One randomized controlled trial suggests that antibiotic treatment for VAT may reduce subsequent VAP rates and ICU mortality while increasing mechanical ventilation-free days [10]. However, in the face of the menacing threat of increased antibiotic resistance, most clinicians exercise caution, balancing potential benefits against the risk of fostering multidrug-resistant organisms [11]. In contrast, VAP management is more clearly defined, typically involving prompt empirical broad-spectrum antibiotics followed by rapid de-escalation and a short treatment course (often 8 days) [10]. Inhaled antibiotics are also used in some cases. Whether these adult-derived strategies can be safely and effectively extrapolated to pediatric patients—particularly those receiving NIV rather than IMV—remains unknown.

Furthermore, while VAP and VAT are well-characterized in invasively ventilated patients, the epidemiology and clinical relevance of lower respiratory tract infections in children managed with NIV are poorly defined. In routine practice, antibiotic initiation in this population is often driven by clinical judgment rather than evidence-based criteria, raising concerns regarding both overtreatment and the emergence of antimicrobial resistance. This lack of pediatric-specific evidence represents a critical knowledge gap and provides the rationale for further investigation into ventilator-associated infections and antibiotic use in children supported with NIV in the PICU.

### Study Objective

The objective of this study was to describe the occurrence and microbiological characteristics of respiratory infections and antibiotic use in children with respiratory failure admitted to PICU stratified by the type of respiratory support provided (oxygen therapy, non-invasive ventilation, or invasive mechanical ventilation).

Given the retrospective observational design, the study was intended to be descriptive and hypothesis-generating, rather than to identify independent risk factors, determine infection incidence rates, or establish causal relationships.

## 2. Results

**Figure 1:**
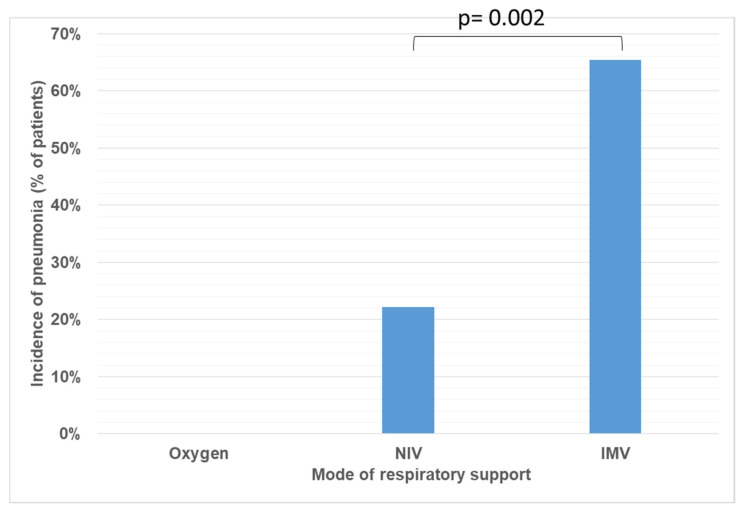
Distribution of infection diagnoses according to respiratory support modality. VAP was diagnosed exclusively in patients receiving IMV. In patients receiving NIV, infectious episodes were classified as lower respiratory tract infections. Bars represent the proportion of patients with pneumonia within each respiratory support group.

**Figure 2:**
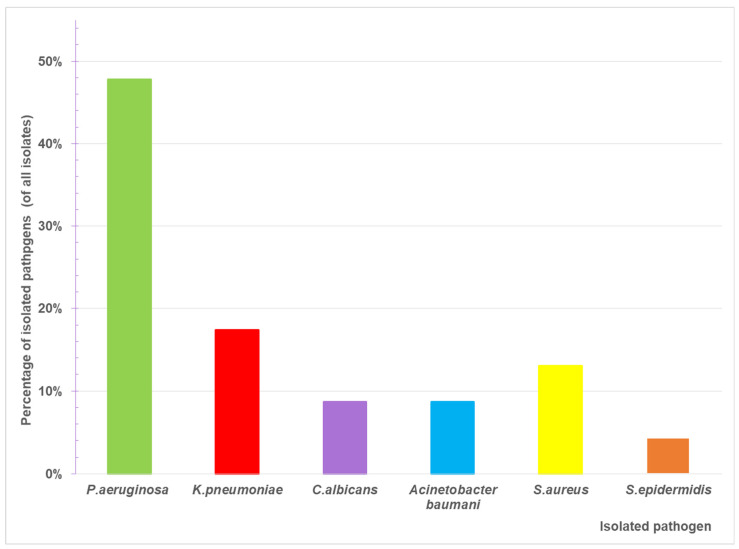
Distribution of isolated pathogens among patients with positive microbiological results. Values represent the percentage of each pathogen among all positive cultures obtained during the study period.

**Figure 3:**
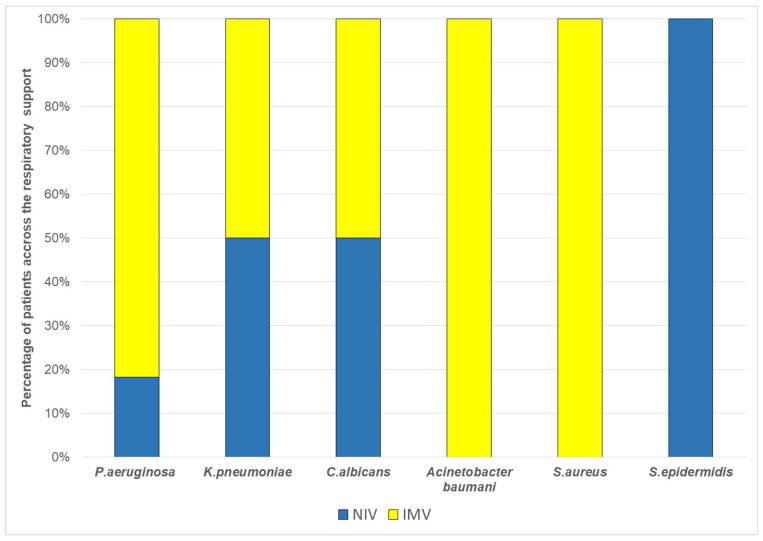
Proportion of patients with each isolated pathogen stratified by respiratory support modality (NIV or IMV).

For a 4-year period (2021–2025), 89 children aged 0 to 18 years (excluding neonatal period) were admitted to the PICU with respiratory failure requiring respiratory support. For analysis, patients were divided into three groups: Group 1 (n = 36) received oxygen via nasal cannula only; Group 2 (n = 27) was treated with NIV; Group 3 (n = 26) required IMV. The main demographic, clinical, and laboratory characteristics of the study population are summarized in Table 1. Patients requiring only oxygen support were statistically younger, and most of them had bronchiolitis. NIV and IMV groups were approximately three years older, with no significant difference between them, and most had underlying chronic metabolic or neuromuscular disorders. No cases of VAT were diagnosed in Groups 2 and 3. However, lower respiratory tract infections were documented in 22.22% of patients receiving NIV and 65.38% of patients receiving IMV (Figure 1). The distribution of infection diagnosis differed significantly between respiratory support groups (Chi-square test with Yates correction: χ^2^ = 7.570, *p* = 0.005); however, this comparison should be interpreted cautiously given the differing group definitions and baseline characteristics between the groups. Additionally, lower respiratory tract infection in NIV patients reflects a broader clinical diagnosis than VAP in the IMV group. The most prevalent isolated pathogen was *Pseudomonas aeruginosa* (48%), followed by *Klebsiella pneumoniae* and *Staphylococcus aureus* (Figure 2). Pathogen distribution also varied—*Staphylococcus aureus* and *Acinetobacter baumannii* were isolated exclusively from children receiving IMV (Figure 3).

**Figure 4:**
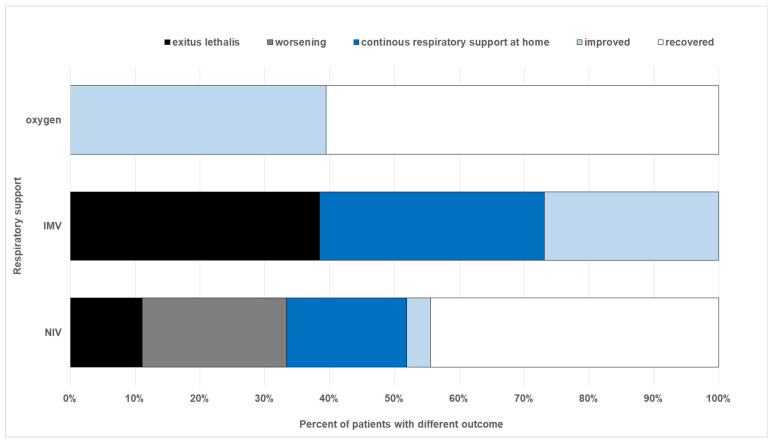
Clinical outcomes according to respiratory support modality. Outcomes are categorized as recovery, partial recovery, or death. Patients initially treated with NIV, who later required IMV, were analyzed in the NIV group, according to the initial respiratory support modality.recovered—fully health ay discharge.

The outcomes differed across groups. All patients in Group 1 achieved partial or complete recovery, while outcomes in Groups 2 and 3 were more variable, ranging from complete recovery to death (Figure 4). A statistically significant difference in the outcomes was observed among all three groups as well as between IMV and NIV groups alone (Chi-square test: χ^2^ = 23.190, *p* < 0.001).

**Figure 5:**
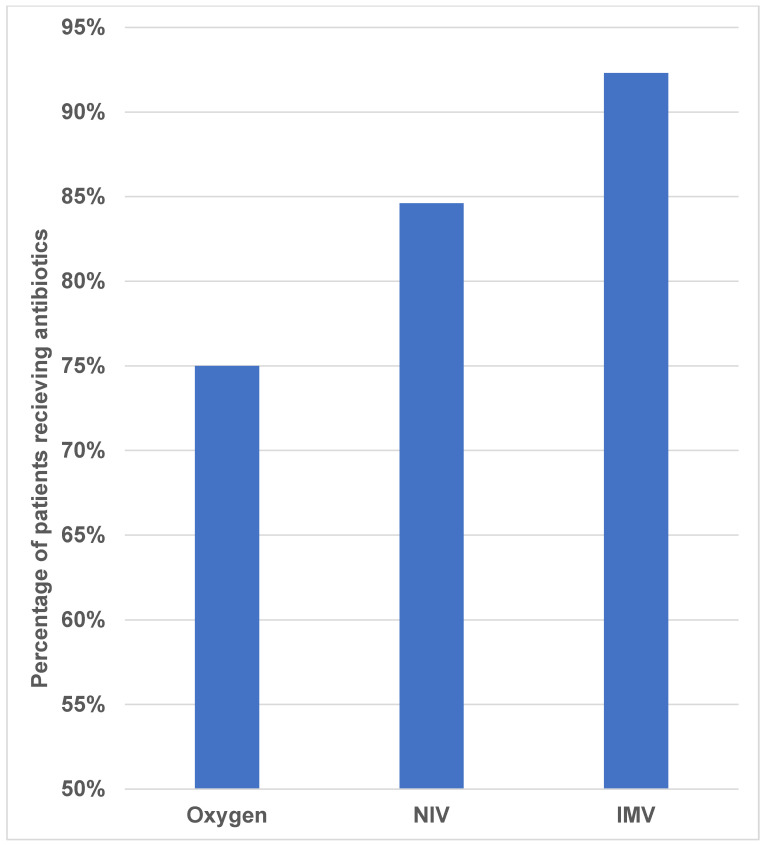
Proportion of patients receiving antibiotic therapy in each respiratory support group.

The analysis of the distribution of patients according to the use of antibiotic treatment in the different groups shows that for all three groups, the largest number of patients were on antibiotic treatment (73 of 88 patients). By group, antibiotics were used in 75.0% of Group 1, 81.5% of Group 2, and 92.3% of Group 3 patients (Figure 5). Notably, all bronchiolitis patients had already received antibiotics prior to ICU admission. The Pearson χ^2^ test showed no statistically significant relationship between the type of respiratory therapy and the administration of antibiotic treatment (χ^2^ = 3.270, df = 2, *p* = 0.195). Correlation analyses were also weak and non-significant (*p* > 0.500), indicating that the choice of respiratory support was not independently associated with antibiotic initiation.

**Figure 6:**
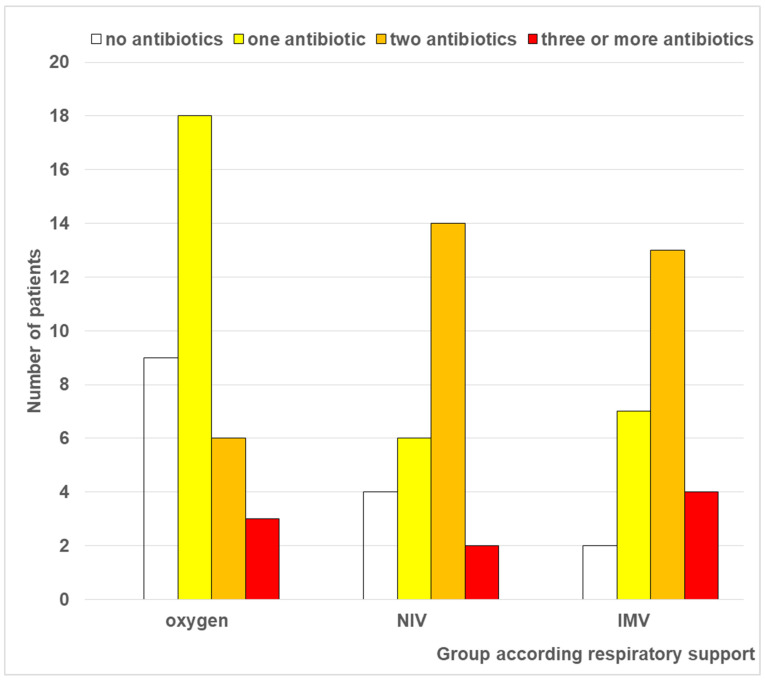
Distribution of patients according to number of antibiotics administered, stratified by respiratory support modality. Group 1—oxygen therapy; Group 2—NIV; Group 3—IMV.

In contrast, comparative analysis of the number of antibiotics administered demonstrated a statistically significant relationship with the type of respiratory support (Figure 6). (Chi-square test: χ^2^ = 14.610, df = 6, *p* = 0.024; ANOVA test: F(2.85) = 4.736, *p* = 0.011; Kruskal–Wallis test: χ^2^ = 9.97, *p* = 0.007). Patients on IMV received a significantly higher number of antibiotics compared to those on oxygen therapy alone (*p* = 0.012–0.013). No difference was found between oxygen therapy and NIV, or between group 2 and group 3. Correlation analysis revealed a moderate positive relationship between the intensity of respiratory support and the number of antibiotics prescribed (Pearson’s R = 0.309, *p* = 0.003; Spearman correlation = 0.329, *p* = 0.002).

## 3. Discussion

This study aimed to describe a single-center experience with antibiotic use in children admitted to the PICU with respiratory failure, stratified by type of respiratory support. As a retrospective observational study with marked population heterogeneity, the findings should be interpreted strictly descriptively. No attempt was made to identify independent predictors of infection or antibiotic exposure, and therefore all between-group comparisons should be considered exploratory and hypothesis-generating only.

NIV is now widely applied in pediatric practice, with reported success rates of 55% and 96%, though outcomes depend heavily on disease-specific and patient-related factors [12]. In our cohort, the most common indication for NIV was underlying neuromuscular disease, characterized by respiratory muscle weakness and impaired airway clearance. Respiratory infections were the second most frequent indication. NIV was successfully applied in cases of acute respiratory failure due to primary pulmonary disease, although severe hypoxemia remains a contraindication for its initiation [13].

The observed differences between groups are likely multifactorial. Patients requiring higher levels of respiratory support were generally older and had a higher prevalence of chronic conditions, which are factors known to be associated with increased disease severity and poorer outcomes. These baseline differences limit direct comparison between respiratory support modalities. In children with neuromuscular disease, prolonged invasive ventilatory support—particularly invasive mechanical ventilation—may compromise natural airway defenses through the presence of an artificial airway, thereby increasing susceptibility to ventilator-associated pneumonia and colonization with more resistant pathogens. In contrast, NIV preserves airway integrity and cough reflexes, which may partially explain the lower incidence of infectious complications observed in this group.

No significant correlations were identified between the type of respiratory support and the antibiotic initiation, length of ICU stay, or patient outcomes in our cohort, supporting the interpretation that antibiotic exposure was primarily influenced by underlying disease severity and infection characteristics rather than by the mode of respiratory support alone.

Based on our clinical experience, antibiotic prescribing patterns in this population reflected several recurring center-specific practice approaches rather than a standardized or evidence-based protocol. These approaches are presented for descriptive purposes only and should not be interpreted as stewardship recommendations.

1. Antibiotics were typically prescribed for confirmed bacterial pneumonia, defined by markedly elevated inflammatory markers and/or positive microbiology; dual therapy with amoxicillin–clavulanic acid and amikacin was used in most cases, while cefepime was preferred in neonates.
2. Antibiotic therapy was commonly discontinued in cases of bronchiolitis with confirmed respiratory syncytial virus infection.
3. Broad-spectrum antibiotics were frequently administered to children with known or suspected underlying immunodeficiency.
4. Children with neuromuscular or metabolic diseases often receive broad-spectrum therapy (e.g., meropenem) with antifungals, reflecting their high infection risk.
5. Vancomycin or linezolid was occasionally added in the presence of a central venous catheter.
6. *Pseudomonas aeruginosa* infections were typically managed with at least dual antibiotic therapy (e.g., colistin and/or meropenem); piperacillin–tazobactam with an aminoglycoside was considered for inadequate response.
7. In children with oncological diseases, antibiotic choice varied by clinical presentation: ceftriaxone for neurological symptoms, piperacillin–tazobactam with metronidazole for intra-abdominal infection, and trimethoprim–sulfamethoxazole for radiologically confirmed pneumonia.

These patterns reflect center-specific clinical practice. The retrospective observational design does not permit causal or comparative conclusions regarding antibiotic effectiveness or appropriateness.

Rising antimicrobial resistance is a growing global concern in pediatric populations [14], highlighting the need for rational antibiotic use and strict adherence to stewardship guidelines in intensive care units [15]. A French multicenter study in pediatric/neonatal ICUs found that adherence to antibiotic treatment protocols was 48.9% and even lower regarding drug choice (27.3%) or duration (26.3%) [15]. A very extensive 2021 review emphasized the need for systematic, pathology-specific ICU antibiotic protocols [16]. Similarly, we observed variability in local practice, highlighting the need for structured stewardship strategies.

As testing and therapies improve (e.g., newborn screening for some metabolic and neuromuscular diseases), more children with chronic conditions will survive and potentially require respiratory support and antibiotics for infections. The controversy surrounding antibiotic use in ICU children requiring respiratory support remains unresolved [16], with limited published data on this growing patient population.

In our study, Gram-negative bacteria were the predominant cause of VAP in invasively ventilated patients. *Klebsiella pneumoniae*, *Acinetobacter baumannii*, *and Pseudomonas aeruginosa* were the most frequent isolates, consistent with prior studies [17,18,19]. This highlights the association between VAP and multidrug-resistant Gram-negative bacteria and underscores the importance of antibiotic stewardship [20].

Consistent with Ortmann et al.’s prospective study [9], we found that antibiotic use (including combinations) did not improve clinical outcomes such as ICU length of stay or need for intubation. While Ortmann’s study focused on a homogenous bronchiolitis population under 2 years, even in our heterogeneous cohort, we found no strong evidence linking antibiotic use to shorter respiratory support or better outcomes.

A recent book on NIV includes a chapter on antibiotics but provides no specific treatment protocol and primarily addresses adult populations with chronic obstructive pulmonary disease, Cystic Fibrosis, or immunosuppression [21]. In the pediatric population, NIV is essential for neuromuscular disease, and may reduce antibiotic needs for respiratory infections in this group [22]. A recent review of neurological patients (including 5115 subjects) emphasized targeted therapy for proven *Pseudomonas* spp. recommending piperacillin-tazobactam if standard therapy fails [23], aligning with our finding of *Pseudomonas aeruginosa* as the leading pathogen. For children requiring long-term ventilation via tracheostomy, Pearce et al. summarize antibiotic strategies, noting the need for randomized clinical trials, as all data comes from retrospective cohorts such as ours. Systemic antimicrobials covering *Pseudomonas* and *Staphylococcus* (e.g., fluoroquinolones, beta-lactams with aminoglycosides) are routinely used [24]. Optimal duration is still unclear. Inhaled antibiotics are an option for long-term therapy [25]. A 2019 scoring system for ventilated children under 3 may help guide decisions. Scores < 2 on day 3 correlate with good outcomes, suggesting antibiotics can be stopped, while scores ≥ 6 indicate a need for prolonged therapy [26].

### Limitations of the Study

The main limitations of this study include its retrospective, single-center design and the relatively small sample size (n = 89). Although the hospital is located in the capital city of Bulgaria, the PICU has a capacity of only six beds and represents one of only three PICUs in Sofia, which may limit the generalizability of the findings.

The heterogeneity between patient groups receiving oxygen therapy, non-invasive ventilation, and invasive mechanical ventilation in terms of age, underlying diagnoses, and disease severity, represents important confounders that were not controlled for through matching or multivariable adjustment As a result, observed differences in infection rates, antibiotic use, or clinical outcomes should not be attributed to the mode of respiratory support itself but are more likely influenced by baseline patient characteristics.

Ventilator-associated pneumonia is conventionally defined in the setting of invasive mechanical ventilation, and its applicability to patients receiving non-invasive ventilation is therefore limited; moreover, differences in microbiological sampling methods may have affected pathogen comparisons across groups. In addition, the analyses were primarily univariate, and the lack of multivariable adjustment for key clinical factors limits causal interpretation of some statistically significant findings.

Prospective, multicenter controlled trials are needed to establish evidence-based guidelines for antibiotic use in children requiring ventilatory support.

## 4. Materials and Methods

### 4.1. Study Design, Participants and Data Collection

We conducted a retrospective study in children admitted with respiratory failure requiring respiratory support (oxygen supplement, NIV or IMV) in the PICU of the University Hospital for Active Treatment of Children’s diseases “Professor Ivan Mitev”, Sofia, Bulgaria, between January, 2021 and February, 2025. The study period from January 2021 to February 2025 was selected because NIV became a standard modality in our PICU starting in January 2021. Selecting this timeframe ensured consistency in clinical practice, data availability, and staff experience, allowing for a more reliable descriptive assessment of respiratory support strategies and associated antibiotic usage

We used electronic medical records of patients admitted to the ICU. The extracted data included demographic characteristics of the patients, type of respiratory failure, clinical diagnosis, and laboratory results at admission and follow-up, prior antibiotic use and subsequent antibiotic use, length of respiratory support, length of stay, and outcome. We divided the patient in three groups according to the respiratory support. The decision to institute the NIV was made by a pediatric ICU physician. NIV was considered as a treatment when the patient presented with respiratory failure, either hypercapnia (PCO2 > 50 mmHg) or hypoxemia (oxygen Saturation < 90%) or both.

### 4.2. Inclusion and Exclusion Criteria

Inclusion criteria: PICU admission during the study period with signs of respiratory failure and hospitalization > 12 h.

Exclusion criteria: PICU stay < 12 h; admission before the study period; age < 28 days (managed in neonatal ICU); Glasgow Coma Scale (GCS) < 8 or altered mental status in previously normal patients, and cardio-circulatory instability. The reason for this exclusion is that impaired consciousness is a recognized absolute contraindication to non-invasive ventilation. In comatose patients, upper airway protective reflexes are compromised, resulting in an unsecured airway and a high risk of aspiration, making NIV unsafe in this population.

**Figure 7:**
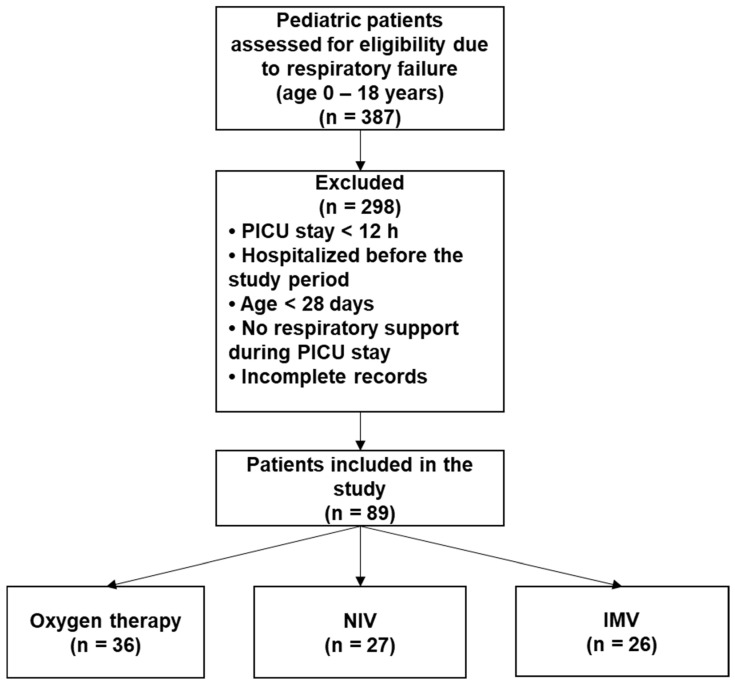
Flowchart diagram illustrating patient selection and inclusion in the study.

Eighty-nine children met all the criteria (Figure 7).

### 4.3. Definitions on Respiratory Infections

VAP and VAT were defined and assessed only in patients IMV via an endotracheal or tracheostomy tube, with VAP diagnosed ≥48 h post-intubation based on clinical signs, laboratory findings, and microbiological criteria [27,28]. VAT was defined by purulent tracheal secretions with positive cultures. In NIV patients, infectious episodes were classified as lower respiratory tract infections based on clinical, laboratory, and available microbiological data [27,29,30].

### 4.4. Microbiological Tests

Respiratory samples were obtained via two approaches using invasive and minimally invasive techniques, as clinically appropriate.

Bronchoalveolar lavage (BAL) is considered the most reliable method [31]; however, it requires either IMV or bronchoscopy. In patients receiving oxygen supplementation or NIV, respiratory specimens were collected using minimally invasive techniques, consisting of upper airway swabs or tracheal aspirates, depending on the level of respiratory support.

### 4.5. Statistical Analyses

Data are presented as median (interquartile range, IQR) or number (percentage). Laboratory parameters reflect values obtained at hospital admission. Patients were grouped according to the highest level of respiratory support received during hospitalization. Group comparisons used the χ^2^ test for categorical variables and the Kruskal–Wallis test for continuous variables; the Mann–Whitney U test was used for length of hospital stay (available for NIV and IMV only). A *p* value < 0.05 was considered statistically significant. Statistical analysis was performed using the Statistical Package for Social Sciences (SPSS version 19).

### 4.6. Ethical Consideration

The study received approval from the Ethics Committee of the Medical University of Sofia, Bulgaria.

## 5. Conclusions

In this single-center retrospective cohort, antibiotic use was common among children requiring PICU respiratory support. Antibiotic exposure appeared to be influenced primarily by underlying disease and clinical severity rather than by the mode of respiratory support itself. VAP occurred exclusively in invasively ventilated patients, while NIV was associated with fewer documented infection-related events.

Given the heterogeneity of the study population and the lack of multivariable adjustment for key confounders, these findings should be interpreted as descriptive and hypothesis-generating. They highlight the need for prospective, multicenter studies using standardized infection definitions and robust analytical methods to define optimal antibiotic strategies for pediatric patients receiving respiratory support. Underlying conditions of the patient appeared to influence the antibiotic selection more strongly than the type of respiratory support. NIV can be an effective alternative to IMV in acute and chronic respiratory failure when applied with appropriate clinical judgment. In patients requiring long-term ventilation, antibiotic therapy—including inhaled formulations—should be individualized based on the microbiological findings and underlying disease to avoid unnecessary exposure in children without clear evidence of bacterial infection.

## Figures and Tables

**Table 1:** Demographic, clinical, and laboratory characteristics of pediatric patients admitted to the PICU with respiratory failure, stratified by highest level of respiratory support received.

Variable	Oxygen Therapy (O_2_)	Non-Invasive Ventilation (NIV)	Invasive Mechanical Ventilation (IMV)
**Participants, *n***	36	27	26
**Sex, *n*** (**%)**			
Male	22 (61.1)	10 (37.0)	13 (50.0)
Female	14 (38.9)	17 (63.0)	13 (50.0)
**Age, years**			
Median (IQR)	0.6 (0.2–3.8)	4.2 (0.6–11.3)	3.0 (0.9–7.5)
**Age groups, *n* (%)**			
0–5 years	30 (83.3)	15 (55.6)	18 (69.2)
6–9 years	3 (8.3)	4 (14.8)	3 (11.5)
10–14 years	1 (2.8)	4 (14.8)	4 (15.4)
15–18 years	2 (5.6)	4 (14.8)	1 (3.8)
**Length of hospital stay, days**			
Median (IQR)	NA	14.5 (7.0–24.0)	18.0 (7.2–32.8)
**Clinical signs and symptoms at admission**			
**Most common presenting features**	Acute bronchiolitis, pneumonia	Pneumonia, neuromuscular disease	Severe pneumonia, sepsis
**Laboratory parameters at admission**			
White blood cell count (×10^9^/L)	12.2 (8.2–15.7)	11.0 (7.2–14.8)	12.4 (8.1–15.0)
Hemoglobin (g/L)	112.5 (102.8–137.0)	113.0 (89.0–128.0)	98.0 (84.5–118.0)
Platelets (×10^9^/L)	363.0 (260.5–442.0)	330.0 (232.0–438.0)	252.0 (183.5–396.5)
Neutrophils (%)	58.1 (43.0–72.2)	56.5 (42.0–76.0)	61.0 (44.0–78.0)
Lymphocytes (%)	30.2 (16.9–44.0)	30.0 (14.0–44.0)	26.0 (12.0–39.0)
C-reactive protein (mg/L)	10.7 (2.4–28.2)	24.0 (7.1–80.0)	39.8 (9.9–133.5)
Procalcitonin (ng/mL)	0.2 (0.1–0.5)	0.3 (0.1–1.4)	0.3 (0.1–2.0)
Erythrocyte sedimentation rate (mm/h)	18.0 (9.0–30.0)	24.0 (12.0–44.0)	30.0 (15.0–59.0)
Sodium (mmol/L)	137.0 (135.0–140.0)	137.0 (134.0–140.0)	137.0 (134.0–141.0)
Potassium (mmol/L)	5.2 (4.6–5.6)	4.5 (3.9–5.1)	4.6 (4.0–5.5)
Creatinine (µmol/L)	28.0 (21.0–41.0)	28.0 (20.0–44.0)	30.0 (23.0–56.0)
Lactate (mmol/L)	1.4 (1.0–2.0)	1.6 (1.1–2.5)	1.8 (1.1–2.8)
Albumin (g/L)	40.7 (38.9–44.0)	38.9 (32.0–44.5)	31.0 (29.3–39.0)
Aspartate aminotransferase (U/L)	46.0 (31.0–66.0)	45.0 (29.0–72.0)	58.0 (33.0–92.0)
Alanine aminotransferase (U/L)	29.0 (18.0–43.0)	26.0 (14.0–45.0)	29.0 (16.0–54.0)

## Data Availability

The raw data supporting the conclusions of this article will be made available by the authors on request.

## Abbreviations

The following abbreviations are used in this manuscript:

BAL	Bronchoalveolar lavage
GCS	Glasgow Coma Scale
ICU	Intensive Care Unit
IMV	Invasive Mechanical Ventilation
IQR	Interquartile Range
LRTI	Lower Respiratory Tract Infection
NIV	Non-Invasive Ventilation
PaCO_2_	Partial Pressure of Carbon Dioxide in Arterial Blood
PICU	Pediatric Intensive Care Unit
RSV	Respiratory Syncytial Virus
VAT	Ventilator-Associated Tracheobronchitis
VAP	Ventilator-Associated Pneumonia
